# Silica coated iron nanoparticles: synthesis, interface control, magnetic and hyperthermia properties†

**DOI:** 10.1039/c8ra06075d

**Published:** 2018-09-17

**Authors:** A. Glaria, S. Soulé, N. Hallali, W.-S. Ojo, M. Mirjolet, G. Fuks, A. Cornejo, J. Allouche, J. C. Dupin, H. Martinez, J. Carrey, B. Chaudret, F. Delpech, S. Lachaize, C. Nayral

**Affiliations:** LPCNO, Université de Toulouse, CNRS, INSA, UPS 135 Avenue de Rangueil 31077 Toulouse France slachaiz@insa-toulouse.fr fdelpech@insa-toulouse.fr cnayral@insa-toulouse.fr; Institut des Sciences Analytiques et de Physico-Chimie pour l’Environnement et les Matériaux, Université de Pau et des Pays de l’Adour, Hélioparc 2 av. Président Angot F-64053 Pau France

## Abstract

This work provides a detailed study on the synthesis and characterization of silica coated iron nanoparticles (NPs) by coupling Transmission Electronic Microscopy (TEM), X-ray Photoelectron Spectroscopy (XPS) and magnetic measurements. Remarkably, iron NPs (of 9 nm of mean diameter) have been embedded in silica without any alteration of the magnetization of the iron cores, thanks to an original protocol of silica coating in non alcoholic medium. Tuning the synthesis parameters (concentration of reactants and choice of solvent), different sizes of Fe@SiO_2_ composites can be obtained with different thicknesses of silica. The magnetization of these objects is fully preserved after 24 h of water exposure thanks to a thick (14 nm) silica layer, opening thus new perspectives for biomedical applications. Hyperthermia measurements have been compared between Fe and Fe@SiO_2_ NPs, evidencing the self-organization of the free Fe NPs when a large amplitude magnetic field is applied. This phenomenon induces an increase of heating power which is precluded when the Fe cores are immobilised in silica. High-frequency hysteresis loop measurements allowed us to observe for the first time the increase of the ferrofluid susceptibility and remanence which are the signature of the formation of Fe NPs chains.

## Abstract 2

A novel method has been developed for the silica coating of iron nanoparticles while preserving the magnetic properties.

## Graphical abstract

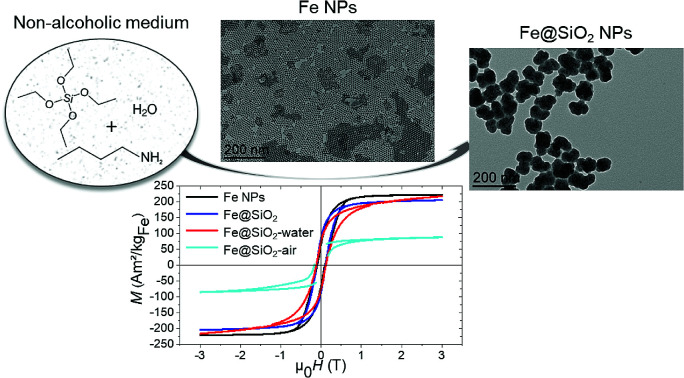

## Introduction

Magnetic nanoparticles (MNPs) have proven to be of wide interest over the last twenty years for applications in catalysis, electronics, biology and medicine.^1–4^ They can be seen on one hand, as holding a static magnetic moment when retaining a bit of information and used for detection or manipulation of proteins, cells, DNA, molecules, catalysts, *etc.*, or on the other hand, as holding a dynamic magnetic moment when perturbing the proton spin relaxation in MRI, or releasing heat under AC magnetic excitation in hyperthermia experiments. Whatever the application, a strict control of their magnetic properties is the key to achieve a high efficiency of the required effect. Chemists and physicists have now deeply increased their knowledge about the relation between the magnetic properties and the size, shape, crystalline structure and surface state of the NPs.^5–8^ In several cases, they also have developed models that allow them to predict what would be the ideal MNP for one particular potential application. However, obtaining the right magnetic properties is not sufficient if the nano-object stability (over oxidation or aggregation in the blood stream for example) and the possibility to integrate it into a more complex structure (self-organized monolayer for data storage, catalyst support, drug carrier, ligand receptor…) are not insured. These ones need to be addressed while preserving the optimized magnetic properties.^9^ The present study takes place in this context. Whereas a lot of development has been done with iron oxide or ferrite MNPs,^10–15^ we have worked on metallic iron MNPs for more than 15 years. Our basic motivation comes from the fact that Fe(0) saturation magnetization *M*_s_ is over twice as high as the ones of maghemite Fe_2_O_3_ and magnetite Fe_3_O_4_ (220 A m^2^ kg^−1^ against 82 A m^2^ kg^−1^ and 92 A m^2^ kg^−1^ respectively). All the magnetic effects “amplitudes” are positively affected by a magnetization increase. For examples, the strength of a nano-magnet linearly increases with *M*_s_, as well as magnetic hyperthermia maximum heating power. In parallel to the MNPs syntheses and characterizations, we have gained a deep understanding on experimental magnetic fluid hyperthermia (MFH) and related theoretical models.^7,8,16^ It has confirmed that metallic iron MNPs are potentially better candidates for MFH than iron oxides ones, provided that we can insure their stability against oxidation. This issue was not limited to MFH applications but also appears in all cases in which these MNPs are subsequently used under oxidizing environments (water, air). To the best of our knowledge, there is no reference in literature that describes a protective coating method of metallic magnetic NPs that fully preserves the original magnetic properties of an oxidizable core. This pushed us to develop a new protection method of metallic iron MNPs. Since we had designed a synthesis protocol of silica NPs in a non-alcoholic medium,^17^ we then intended to adapt it to coat metallic iron NPs and obtain Fe@SiO_2_ nano-objects free from oxidation. Silica is a versatile material for an additional inorganic outer-layer and its functionalization is well developed for a wide variety of application fields.^18,19^ However, the two main protocols described so far for the formation of silica around an inorganic core are not suitable to preserve the oxidation state of our metallic iron MNPs. They are: (i) the so-called Stöber process where tetraethyl orthosilicate (TEOS) is hydrolyzed in an ethanolic medium under addition of aqueous ammonia and (ii) a water-in-oil micro-emulsion using principally a non-ionic surfactant promoting the TEOS hydrolysis inside a micelle formed around the NPs core dispersed in an organic solvent.^20–22^ Therefore, the development of a new silica coating procedure, truly preserving the magnetic integrity of the metallic core, was a necessary step before such high magnetization MNPs could be used in various applications. An additional challenge was to keep the control of the overall size of the nano-objects, and hence of the shell thickness. As an example, particles over 100 nm are rapidly cleared by the liver and spleen, thus limiting their use in biomedical applications like drug delivery or hyperthermia.

We report here our results on this novel silica coating method. We explored the influence of key parameters such as the solvent and the reactants concentrations. We particularly paid attention to characterize the nature of the interface between Fe(0) and SiO_2_ to evaluate the oxidation due to the coating step. The magnetic properties have been studied and compared to the ones of the pristine Fe NPs and then followed by magnetic hyperthermia measurements. Finally, the impact of the exposition to air and to water has been evaluated.

## Experimental

### Chemicals

All the preparations and syntheses are performed under an inert atmosphere of argon either in a glove box or in Fisher-Porter bottles. TEOS and 1-butylamine were purchased from Alfa Aesar. Tetrahydrofuran (THF) and dimethoxyethane (DME) were supplied by Carlo Erba and purified using an Innovative Technology system and then degassed through argon bubbling. Deionised (DI) H_2_O was obtained using a Veolia Water STI Aquadem purifier giving water with a 18 MΩ cm resistivity. Iron NPs were purchased from NanoMePS. All the chemicals were used without further treatment.

### Synthesis of Fe@SiO_2_ NPs

In a typical procedure 450 μL (2 mmol) of TEOS and 98 μL (1 mmol) of 1-butylamine were mixed in a Fisher Porter bottle with 18.5 mL of THF. Then 72 μL (4 mmol) of DI H_2_O were added under argon and the solution was magnetically stirred for 4 h at 70 °C. Meanwhile 66 mg of Fe NPs were dispersed in 3.5 mL of THF and sonicated for 20 min at 40 °C. Finally, the iron NPs were added under argon to the TEOS solution and the reaction proceeded for 7 days at 70 °C. The black powder was centrifuged (17 000 rpm – 25 202 G/15 min) and washed with THF before drying under vacuum overnight. The resulting material was stored in an argon filled glove-box until further characterization.

### Characterization

#### XPS

XPS measurements were performed on a Thermo K-alpha spectrometer with a hemispherical analyzer and a microfocused (400 μm diameter microspot) monochromated radiation (Al Kα, 1486.6 eV) operating at 72 W under a residual pressure of 1 × 10^−9^ mbar. The XPS spectrometer was directly connected to an argon dry box in order to avoid any moisture and air exposure of the samples. The pass energy was set to 20 eV. Charging effects were compensated by the use of a dual beam charge neutralization system (low energy electrons and Ar^+^ ions), which had the unique ability to provide consistent charge compensation. All spectra were energy calibrated by using the hydrocarbon peak at a binding energy of 285.0 eV. Spectra were mathematically fitted with Casa XPS software© using a least squares algorithm and a nonlinear Shirley-type background.^23^ The fitting peaks of the experimental curves were defined by a combination of Gaussian (70%) and Lorentzian (30%) distributions except for iron metal which was fitted with an asymmetric peak shape (LA(1.2,4.8,3)) as Biesinger *et al.*^24^ Quantification was performed on the basis of Scofield's relative sensitivity factors.^25^

#### TEM

Samples for TEM analysis were prepared by deposition of a drop of a diluted solution on an amorphous carbon-coated copper grid. Low-resolution images were obtained with a JEOL-1400 microscope operating at 120 kV.

#### VSM

Powders were sealed in a VSM capsule under argon atmosphere and analyzed using a VSM PPMS – Quantum Design system. Hysteresis loops were performed in a −3/+3 Tesla range at 300 K and at 4 K after field cooling.

#### Elemental analysis

Fe NPs were analyzed after a full oxidation/reduction process using an TGA apparatus. All Fe@SiO_2_ samples were sent to Actlabs (Ancaster. ON) where 20 mg were analyzed using the instrumental neutron activation analysis (INAA) technique which gave % Fe with a 0.05% accuracy.

#### Magnetic hyperthermia

Temperature measurements for specific absorption rate (SAR) evaluation was carried out by placing a Schlenk containing between 12 and 31 mg of powder, and 0.5 mL of mesitylene in a calorimeter with 1.5 mL of water. This water played the role of heat transfer liquid, the temperature of which was measured during the experiment. The calorimeter was placed inside a commercial coil (Fives Celes) and exposed to an alternating magnetic field (AMF) of 93 kHz with an amplitude ranging from 5 mT to 50 mT. Temperature measurement was performed by putting two optical probes (Reflex Neoptix 4) at the top and the bottom of the calorimeter. The magnetic field exposure time was fixed to 100 s. The temperature rise at the end of the magnetic field application was measured after shaking the calorimeter to ensure the temperature homogeneity and never exceeded 15 K. SAR values of the sample were calculated using the following expression:
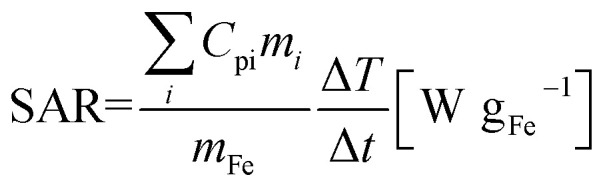
where *C*_pi_ and *m*_*i*_ are specific heat capacity and mass for each component respectively and *m*_Fe_ is the mass of the pure iron. Because the calorimeter is not perfectly adiabatic, heat losses make measured temperature values lower than if calorimeter was adiabatic. After calibration experiments, we found that for an AMF application time of 100 s, SAR values have to be multiplied by a factor 1.3 to correct them from calorimeter losses.

#### High frequency hysteresis loop measurements

Hysteresis loop measurements were performed using a home-made coil, described in ref. 26 and generating an AMF with a variable amplitude at 50 kHz. The essential elements of this setup were pickup coils that recorded induced electromotive forces according to Faraday–Maxwell law of magnetic induction.^26^ The amplitude of the alternating magnetic field was obtained using 
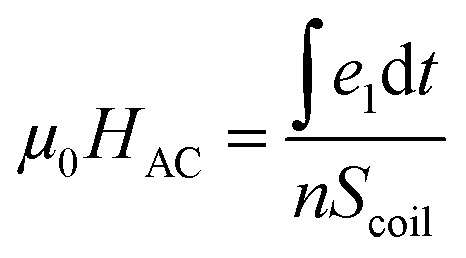
 with *e*_1_ the voltage of the empty coil. The magnetization per unit mass *M* of the NPs was obtained using 
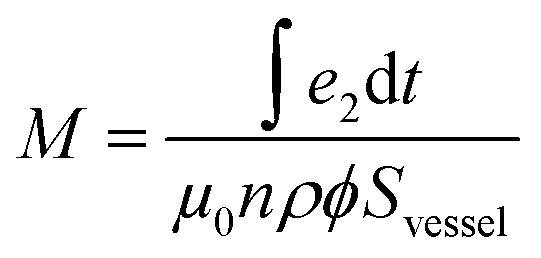
, with *S*_vessel_ the surface of a section of the vessel containing the colloidal solution, *ϕ* the volume concentration of the sample, *ρ* the magnetic NPs density and *e*_2_ the voltage at the terminal of the two coils in series.

In the case of Fe@SiO_2_ samples, NPs settled down fastly thus *ϕ* could only be estimated, but not precisely known. The volume concentration was estimated by measuring the NP powder height inside the Schlenk – which permits to evaluate the volume of material – and then to divide it by the iron mass. When using this estimation, discrepancy between the magnetic method and the calorimetric one could reach a factor as large as 2.3. Therefore, to offset the difference between calculated magnetization values and the real ones we calculated the area (*A*_loop_) of the estimated hysteresis loop as a function of AMF amplitude value (*μ*_0_*H*_max_). We compared this curve to the one corresponding to the specific losses obtained from temperature measurements (A_Temp_). The *A*_loop_ (*μ*_0_*H*_max_) curve was multiplied by a corrective factor *x*_corr_ (ranging from 0.7 to 2.3) so as to obtain a good correspondence with the *A*_Temp_ (*μ*_0_*H*_max_) curve (see ESI†-Fig. 4). This correcting factor was then applied to the magnetic hysteresis loops. After this treatment, the magnetization curves are expected to represent quantitatively the high-frequency magnetic properties of the samples.

## Results and discussion

### Synthesis of Fe@SiO_2_ NPs

**Fig. 1:**
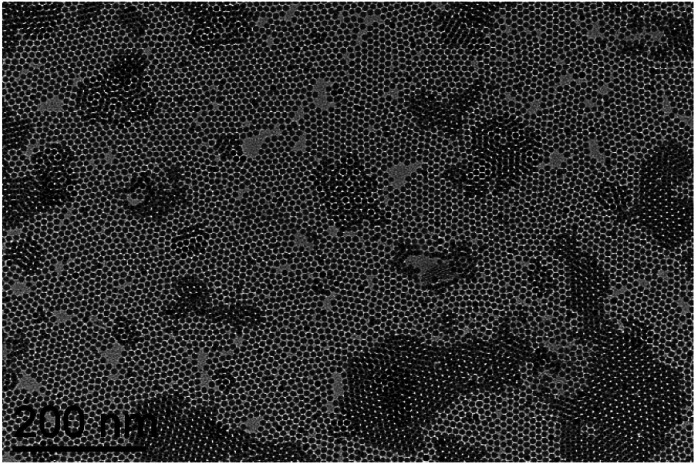
TEM picture of pristine Fe NPs.

For the synthesis of the Fe@SiO_2_ NPs we have used 9 nm Fe NPs as a core material (Fig. 1, ESI†-Fig. 1). These nanomaterials are stabilized by a mixture of palmitic acid and hexadecyl amine, easily dispersible in organic solvents and with magnetic properties corresponding to the ones of the bulk material (*vide infra*). We chose to work with the 9 nm iron cores since their high dispersion in THF insures the homogeneity of the suspension and avoids the presence of large agglomerates.

The metallic NPs were then used as seeds for the growth of the silica material which proceeded over a few days once introduced in the reaction medium. The growth of the silica shell is based on a protocol derived from our previous study on silica formation in a non-alcoholic medium.^17^ The hydrolysis and the condensation of 1 equivalent of tetraethyl orthosilicate (TEOS) occur in an organic solvent (tetrahydrofuran (THF) or dimethoxyethane (DME)), using 1-butylamine (BA) as a catalyst and 2 equivalents of water (minimum amount to allow the silica formation while avoiding an excess of water in the medium). In these conditions, the water exposure of iron NPs is minimized and the kinetic of silica formation is significantly slowed down (compared to classical Stöber protocols). The amine plays also the role of stabilizing agent during and after the synthesis by interacting with the silica surface. The formation of the silica NPs occurs by a slow release in solution of spherical nano-objects from a large silicated network. The coating process has been optimized by playing with the concentration of reactants and with the choice of the solvent, which appear as two levers to control the rate of silica formation. The influence of concentration has been studied in THF and the effect of the solvent has been evaluated by using DME instead of THF. Thus, a set of four samples, as reported in Table 1, is presented here and has been fully characterized, using Transmission Electronic Microscopy (TEM), elemental analysis, magnetic measurements and XPS.

**Table 1:** Experimental conditions used for this study

Sample	Experimental conditions						Remark
	Molar ratios			Time (days)	Solvent	[TEOS] (mol L^−1^)	
	TEOS	BA	H_2_O				
1	2	1	4	7	THF	0.09	Standard
2	2	1	4	7	DME	0.09	Solvent effect
3	1	0.5	2	7	THF	0.045	Concentration effect
4	2	1	4	2	DME	0.09	Time effect

**Fig. 2:**
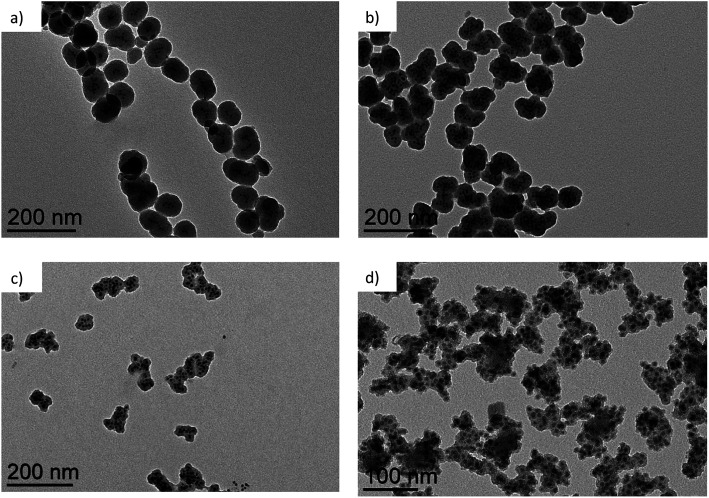
TEM pictures of (a) sample 1, (b) sample 2 and (c) sample 3 after 7 days of reaction and (d) sample 4 after 2 days of reaction.

The TEM pictures of the obtained hybrid NPs (Fe@SiO_2_) (samples 1, 2, 3, 4) are reported in Fig. 2 showing the formation of a silica shell surrounding the iron cores. It is worth noting that, whatever the conditions, no uncoated iron core is observed. The standard procedure, used for sample 1, leads to spheroidal hybrid NPs with a size of 79 (12) nm. For a same reaction time of 7 days, depending on the experimental conditions the final size of the Fe@SiO_2_ NPs can be tuned. It is increased to 89 (14) nm when using DME instead of THF (sample 2) and decreased to 67 (11) nm when reducing the concentration of the reactants compared to the standard protocol (sample 3). These results are in good agreement with the study reported for silica particles alone which showed the same tendency when playing on these two parameters. Here, the introduction of a seed material in the medium did not drastically modify the hydrolysis/condensation process.

To analyze the different steps of the coating, aliquots have been harvested during the reaction (see Table 2). For sample 1, we observed the formation of individualized particles right after day 2 with a large amount of aggregates (micron-size) still present. While the size of the hybrid NPs increased over the days, the number of aggregates was reduced for a complete disappearance at day 7. This tendency was also noted for sample 2 but more pronounced with respect to the increase rate of the NPs size and the disappearance of the aggregates. Indeed, the first hybrid NPs were observable after 48 hours with a size at this stage already approaching the final one obtained using the standard procedure. Then over the days, the NPs population tends to be more homogeneous, with a slight increase in size (12%). When the concentration of the reactants was reduced, *i.e.* sample 3, the reaction was slowed down so that the uncoated Fe NPs were still observable at day 2. Then, aggregates were formed embedding the iron cores and giving rise at day 7 to hybrid NPs with a size of *ca.* 67 nm, nearly 12 nm smaller than the ones obtained in the standard conditions. Nevertheless, these smaller hybrid NPs were still accompanied by a few aggregates. Assuming that the hydrolysis/condensation formation process followed the one described for silica nanomaterials alone, it seems to indicate that the release of hybrid NPs was not yet finished. In the case of sample 4, the use of DME favors a higher rate of formation of the silica and by limiting the time of reaction at 2 days, the silica thickness around the iron cores can be restrained and is then only about 4–5 nm. Individualized hybrid particles are not yet a majority and mainly undefined silica aggregates are observed. Interestingly depending on the experimental conditions the number of Fe NPs encapsulated can also be varied (see Table 2). For sample 1, the number of Fe NPs remained relatively low with an average value of 5 Fe NPs per final hybrid NP. When the solvent was changed for DME, which is prone to increase the reaction rate, the number of Fe NPs was more than doubled up to an average value of 13. However, the iron contents of samples 1 and 2 continue to be quite similar (28.9% for sample 1 and 27.4% for sample 2). For sample 3, the slow reaction process seemed to promote also a higher number of encapsulated NPs closed to the one observed for sample 2, but here the iron content is doubled (57.2%).

**Table 2:** Size analysis of the TEM pictures for the different samples, collected during the reaction. “*d*_TEM,*x*d_” refers to the mean diameter (in nm) of the Fe@SiO_2_ NPs for the sample collected after *x* days of reaction. “agg.” stands for micron-size aggregates. Values given as *x*_c_ (*σ*) using Gaussian fits. The penultimate column indicates the mean number of Fe NPs embedded by silica NP. The last content indicates the weight content of iron in the sample

Sample	*d*_TEM,1d_	*d*_TEM,2d_	*d*_TEM,3d_	*d*_TEM,4d_	*d*_TEM,7d_	Nbr. of Fe NPs	% Fe
1	agg.	53 (12) agg.	65 (14) agg.	68 (12) agg.	79 (12)	5 (3)	28.9
2	agg.	agg.	74 (12) agg.	70 (10), 95 (14)	89 (14)	13 (7)	27.4
3	Fe NPs	Fe NPs	agg.	62 (12) agg.	67 (11) agg.	11 (6)	57.2
4	agg.	agg.	—	—	—	—	35.0

### Magnetic and XPS characterizations

After the coating of the different samples, the magnetic properties were measured by means of hysteresis loops performed at 4 K. These values were compared to the ones of the pure iron NPs used as a core material (to be fully comparable, the magnetization values are expressed per kg of iron). In parallel, XPS which is one of the most suitable methods to characterize the surface of NPs, was used to probe the interface in the core–shell nanosystem. However, considering the XPS depth analysis (around 5 nm), only core–shell NPs with a relatively thin shell give an access to the interface (only sample 4 in this study). The transition metal 2p XPS spectra and more particularly the analysis of mixed iron oxide systems (Fe(ii)/Fe(iii)) is complex because of peak asymmetries, multiplet splitting, shake-up satellite structure and overlapping binding energies.^24^ In this work, the experimental envelope of the Fe2p spectra is reported without any fitting procedure except for metallic iron which presents an asymmetric shape in agreement with literature ref. 24.

#### Pristine Fe NPs

**Fig. 3:**
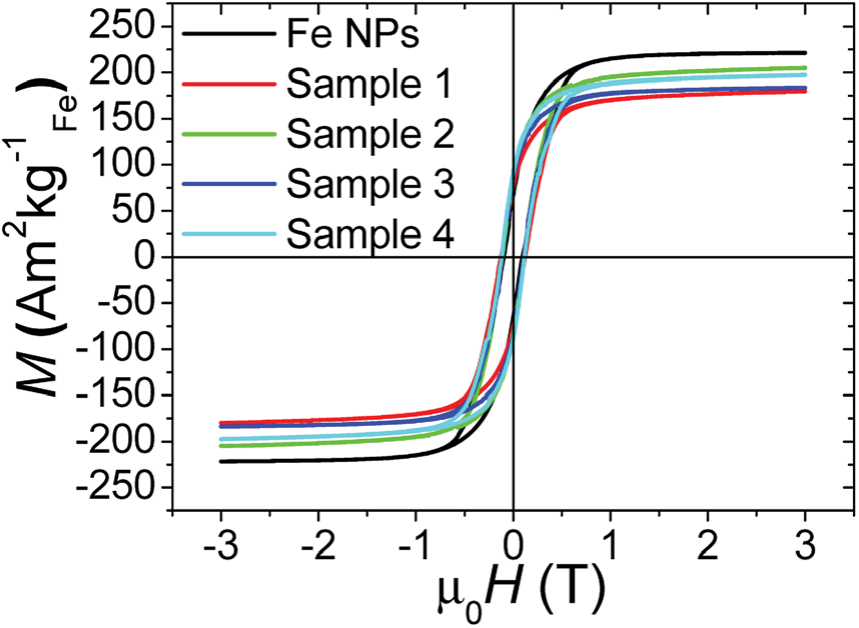
Hysteresis loops of the different samples reported in Table 3 and performed at 4 K.

The saturation magnetization (*M*_s_) of the pure iron NPs which are used as a core material, is found equal to the bulk iron one (see Table 3 and Fig. 3).

**Table 3:** Magnetic measurements performed on the different samples and compared to the pristine Fe NPs. Uncertainty on magnetization values results from the propagation of the uncertainty on the weight content of iron measured in each sample (see Table 2)

Sample	*M*_s_ (A m^2^ kg_Fe_^−1^)	*M*_R_ (A m^2^ kg_Fe_^−1^)	|Δ*μ*_0_*H*| (mT)
Fe NPs	221 (3)	68 (1)	1
1	180 (31)	76 (13)	4
2	205 (37)	85 (15)	6
2-Air	88 (12)	50 (7)	68
2-Water	190–217	75 (10)	0
3	184 (16)	85 (7)	4
4	197 (28)	90 (12)	1
4-Air	86 (12)	69 (9)	34
4-Water	163–191	52 (7)	6

**Fig. 4:**
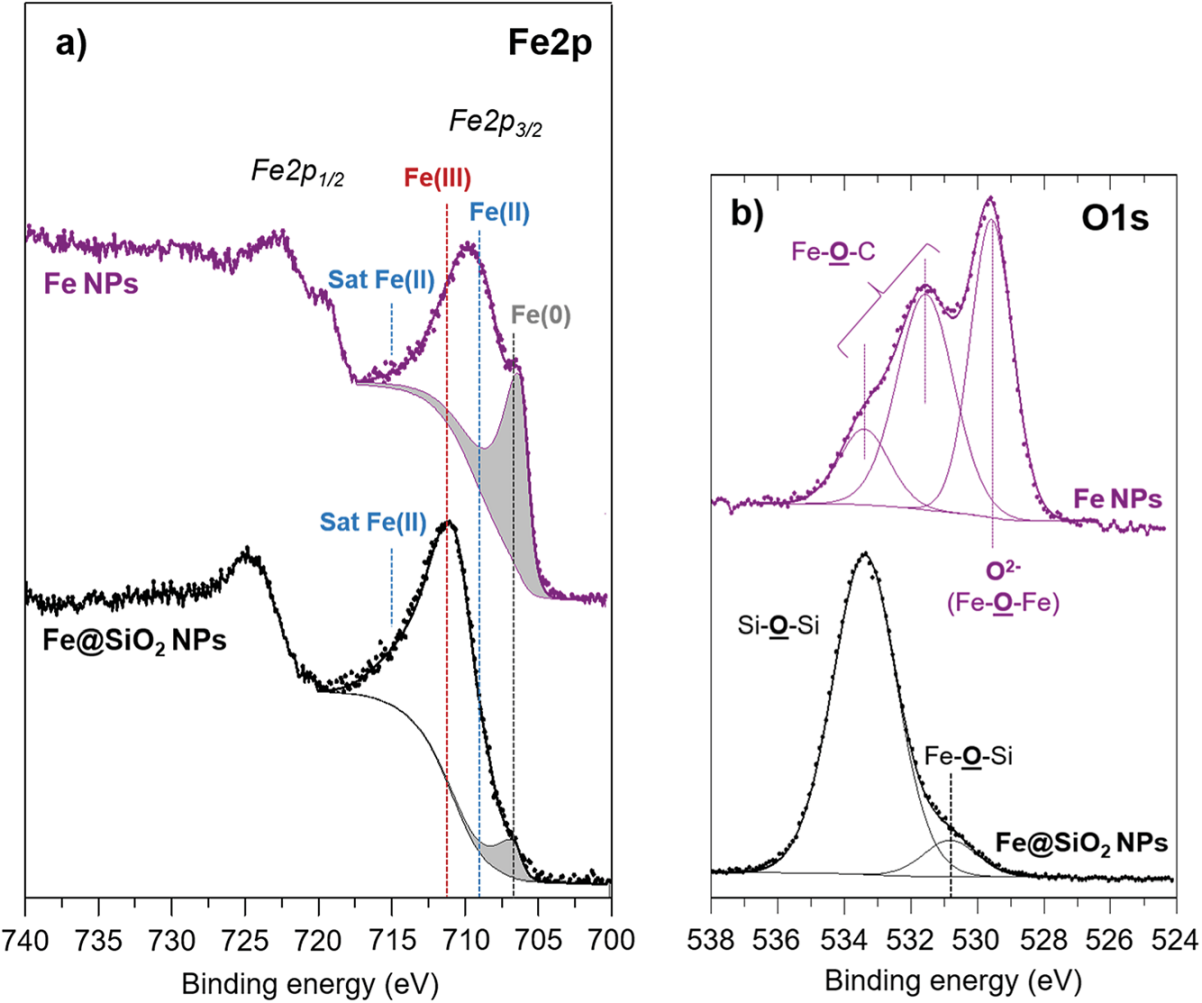
(a) Fe2p and (b) O1s XPS core peaks of Fe and Fe@SiO_2_ NPs.

The Fe2p_3/2_ peak on the Fe2p spectrum of Fe NPs (Fig. 4a) clearly evidences the presence of iron in the oxidation states 0, +II and +III located respectively around 707 eV, 709 eV and 711 eV. Note that the peak also displays the satellite structure of Fe(ii) around 715 eV, increasing the peak asymmetry towards the high binding energies. On the contrary, the shake-up satellite of Fe(iii), expected at 8.5 eV from the Fe2p_3/2_ component, is not clearly identified because it overlaps with the Fe2p_1/2_ components (Fe(0) and Fe(ii)). The deconvolution of iron metal enables to estimate the relative proportion of Fe(0) and of Fe(ii/iii), thus at the surface the estimated Fe(ii/iii)/Fe(0) ratio is 1.5 (Table 4). The O1s core peak of pure iron NPs displays three components (Fig. 4b). The main peak at 529.6 eV (10.0 at%) corresponds to Fe–O–Fe bonds. The two other components located at 531.5 eV (10.1 at%) and 533.4 eV (3.1 at%) can be attributed respectively to Fe–O–C (carboxylate ligand) ‘bidentate’ environments and ‘monodentate’ in accordance with the C–O component at 286.3 eV (10.4 at%) and O=C–O (288.7 eV, 4.2 at%) on the C1s spectrum.^27^ These results clearly point out that at the surface of the NPs (considering the XPS depth analysis), a part of oxidized iron atoms (Fe–O) is detected in different forms, Fe–O–C due to surface carboxylates ligands as well as Fe–O–Fe. However, as shown earlier, these atoms do not induce any detectable effect on the magnetic properties. XPS quantitative analyses (Table 5) also reveal an important atomic percentage of carbon (66.7% at) in agreement with the presence of ligands (palmitic acid and hexadecylamine) at the NPs surface.

**Table 4:** Relative proportions of Fe(0) and Fe(ii/iii), Fe(ii/iii)/Fe(0) ratio calculated from XPS analyses for Fe core, Fe@SiO_2_ NPs and Fe@SiO_2_ NPs exposed to air and water

Sample	Fe (0) (rel. %)	Fe(ii/iii) (rel. %)	Fe(ii/iii)/Fe(0)
Fe NPs	40.8	59.2	1.5
4	7.9	92.1	11.7
4-Air	5.5	94.5	17.2
4-Water	—	100	—

**Table 5:** XPS binding energies (BE), Full Width at Half Maximum (FWHM) and atomic percentages of C1s, O1s, Fe2p, Si2p and N1s core peaks for Fe NPs and Fe@SiO_2_

	Fe NPs		Sample 4	
	BE (eV) (FWHM (eV))	At%	BE (eV) (FWHM (eV))	At%
C1s	285.0 (1.3)	52.1	285.0 (1.6)	11.1
	286.3 (1.7)	10.4	286.3 (1.8)	4.9
	288.7 (1.6)	4.2	288.6 (1.6)	1.6
Total at%		66.7		17.6
O1s	529.6 (1.4)	10.0	530.8 (1.9)	5.3
	531.5 (2.0)	10.1	533.4 (2.3)	53.4
	533.4 (1.9)	3.1		
Total at%		23.2		58.7
Fe2p_3/2_(Fe(0)	706.3	4.1	706.8	0.2
Fe2p_3/2_ (Fe(ii/iii))		6.0		2.8
Total at%		10.1		3.0
Si2p_3/2–1/2_	—	—	103.9–104.5 (2.1–2.1)	20.0
N1s	—	—		0.7

#### Silica coated Fe NPs

The saturation magnetization values obtained for the samples coated in THF, *i.e.* 1 and 3, were respectively of 180 ± 31 and 184 ± 16 A m^2^ kg_Fe_^−1^ and represent on average 81% of the magnetization of the pristine Fe NPs. Associated with no significant shift in the hysteresis loop (Table 3, Fig. 3), these results suggest that a very thin layer of iron oxide appeared but that it does not contain antiferromagnetic coupling at the interface between iron oxide species and iron core. When coating was performed in DME (samples 2 and 4), the final saturation magnetization was very close to the bulk one with a value of 205 ± 37 A m^2^ kg_Fe_^−1^, on average 93% of the initial value. Again, no shift of the hysteresis loop was observed demonstrating that the properties were preserved (Table 3, Fig. 3).

**Fig. 5:**
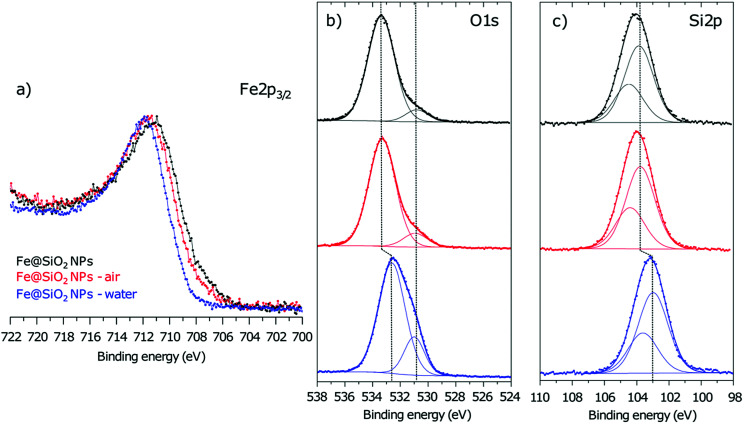
(a) Fe2p, (b) O1s and (c) Si2p XPS core peaks of Fe@SiO_2_ NPs and Fe@SiO_2_ NPs exposed to air and water.

On sample 4, XPS quantitative analysis (Table 5) reveals the prevalence of silicon (20.0 at%) and oxygen (58.7 at%) but also the presence of carbon (17.6 at%) and nitrogen (0.7 at%) due to amine surface ligands. Due to the XPS depth analysis (which is around 5 nm, while 4–5 nm is the silica thickness estimated from TEM pictures on sample 4), the total atomic percentage of iron deduced from XPS analysis is low, *i.e.* 3.0 at%. However, we can notice the shift of the envelope maximum of the Fe2p_3/2_ peak towards high binding energies after the silica coating (Fig. 4a) which reveals the further oxidation at the interface with the silica shell. Thus, the Fe(ii/iii)/Fe(0) ratio is 11.7 for Fe@SiO_2_ compound *versus* 1.5 for iron NPs. The O1s spectrum (Fig. 4b) displays a component at 530.8 eV (5.3 at%) attributed to Fe–O–Si bonds characteristic of the interface. Another component is evidenced at 533.4 eV assigned to SiO_2_ environment, in agreement with the Si2p_3/2_ core peak located at 103.9 eV (Fig. 5c). These results are in agreement with the location of a thin iron oxide layer between the iron core and the silica shell, as detected by the magnetic characterization.

To evaluate the air and water stability of the hybrid NPs, we have chosen to compare the behaviors of two different types of silica shells: sample 2, which exhibits large silica shells around the iron cores (thickness of the silica layer about 14 nm) and sample 4, which presents thinner silica layers (about 4–5 nm of thickness). We thus exposed during 24 h two different aliquots of each sample (2 and 4) to the different media (air and water) and measure the resulting magnetic properties.

#### Air exposure

**Fig. 6:**
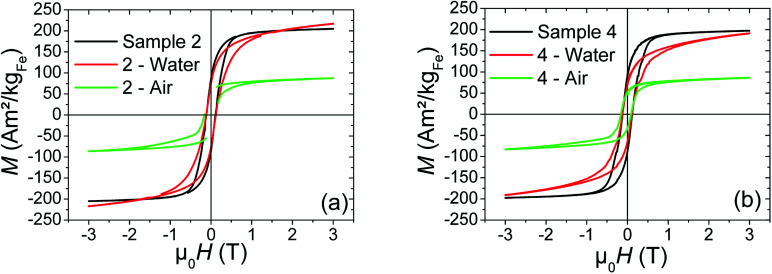
Hysteresis loops performed at 4 K of (a) sample 2, and (b) sample 4, before (black curve) and after exposure to air (green curve) or water (red curve) during 24 h.

In case of the both samples (2 and 4), the results indicated that air exposure drastically affected the magnetic properties, reducing the *M*_s_ down to on average 43% of its initial value (Table 3, Fig. 6). This *M*_s_ decrease and the shift of the hysteresis loop (34 mT) are consistent with an oxidation of the metallic cores. XPS measurements on the sample 4 (the shell of sample 2 being too thick to probe the Fe/SiO_2_, this sample has not been analyzed) show that, after air exposure, the O1s core peak (Fig. 5b) and the Si2p spectrum (Fig. 5c) are not modified. However, the Fe2p_3/2_ peak maximum shifts slightly towards high binding energies (+0.4 eV) (Fig. 5a) and the Fe(ii/iii)/Fe(0) ratio increases from 11.7 (for Fe@SiO_2_ NPs) to 17.2 (Table 4) in agreement with an increase of the thickness of the oxide layer. We note that this evolution of the Fe(ii/iii)/Fe(0) ratio with air exposure is much more significant in the case of Fe NPs (ESI†-Fig. 2 and Table 1) demonstrating the role of the silica coating against oxidation, even if it is not sufficient to fully preserve the magnetic properties.

#### Water exposure

The exposition to water, in the case of sample 4, leads to only a moderate decrease of *M*_s_. One can note that the loops is magnetically unsaturated from 163 to 191 A m^2^ kg_Fe_^−1^. Also, the shift of hysteresis loop (6 mT) is much smaller than the one measured on the air-exposed sample (34 mT), indicating a lower degree of oxidation of the iron compared to air exposure (Table 3). For sample 2, *M*_s_ remains remarkably very close to the one of the unexposed sample, with no shift of the hysteresis loop (Table 3).

Whatever the silica shell thickness (samples 2 and 4), unlike before water exposure, hysteresis loops (Fig. 6) do not saturate at high field. This phenomenon is consistent with the formation of paramagnetic species, such as electrically insulated Fe(ii) and/or Fe(iii). Their proportion (%_para_), and their contribution (*M*_para_) on the magnetic signal measured by VSM (*M*_VSM_) at 4 K, was determined using the following expression:*M*_VSM_ = %_para_*M*_para_ + %_ferro_*M*_ferro_ with %_para_ + %_ferro_ = 1

**Fig. 7:**
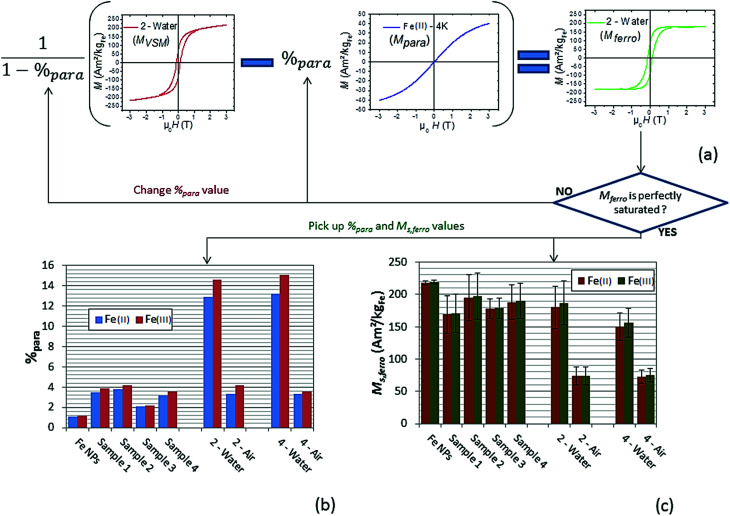
(a) Illustration of the method used and based on *M*_ferro_ formula to determine the paramagnetic contribution %_para_ and *M*_s,ferro_ value. *M*_para_ is obtained theoretically using the Brillouin function applied for magnetically independent Fe(ii) or Fe(iii) species (see ESI† for details of the calculation). This method is illustrated on sample 2 after water exposure and %_para_ is determined to obtain a perfect saturated hysteresis loop (*M*_ferro_) considering Fe(ii) as paramagnetic species. (b) and (c) Paramagnetic contribution %_para_ and *M*_s,ferro_ value obtained for each sample considering either Fe(ii) or Fe(iii) (the non-zero value of %_para_ for NPs Fe sample is not significant but could be linked to a slight spin canting of Fe(0) surface spins).

*M*_ferro_ is the ferromagnetic contribution to the magnetic signal 
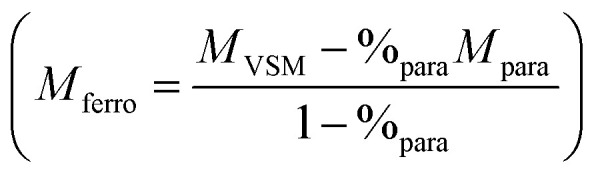
. The saturation value of the *M*_ferro_ signal is called *M*_s_,_ferro_. We assumed that the paramagnetic magnetization *M*_para_ comes from magnetically independent Fe(ii) or Fe(iii) species (see ESI† for details of the calculation). The analysis process is illustrated in Fig. 7a. The paramagnetic contribution can be equally well fitted assuming segregated Fe(ii) or Fe(iii) species, so the shape of the hysteresis loop does not allow us to discriminate between the two. Depending on the type of species assumed, the value of %_para_ is slightly different, but the trends are similar. Here, the effect of water exposure causes a significant increase in the paramagnetic species proportion, going from ∼3% to ∼14% (see Fig. 7b), identically for samples 2 and 4. On the contrary, the fall of *M*_s_,_ferro_ is less important for sample 2 than for sample 4 (Fig. 7c).

XPS analysis of sample 4 after water exposure shows a drastic modification of the silica shell (Fig. 5b and c, ESI†-Fig. 3). The chemical shift of −0.8 eV of both Si2p(SiO_2_) and O(SiO_2_) is consistent with a chemical modification of the silica shell leading to less condensed species.^28^ Besides, the relative intensity of the O1s component at 530.9 eV increases significantly (Fig. 5b) and can be attributed to the formation of FeOSi which is coherent with the apparition of Fe(ii/iii) paramagnetic species evidenced by magnetic measurements. A reasonable hypothesis could be the formation of iron silicate species by diffusion of iron into the less condensed silica shell. In this case, the chemical modification of the silica precludes the direct interpretation of the Fe2p_3/2_ peak shifts, that we will not discuss, since the probed zones (before and after water exposure) are not comparable anymore.

To sum-up, the consistency between XPS and VSM measurements allows us to draw up the following evolution scheme concerning the Fe NPs and Fe@SiO_2_ NPs: (i) before the synthesis of the silica shell, the Fe NPs are essentially composed of Fe(0). A small percentage of iron atoms have a higher oxidation degree, such as Fe(ii) and/or Fe(iii). (ii) After the silica shell growth, a slight layer of iron oxide appears and the magnetization of the iron cores decreases slightly. This result is not surprising with an interface necessarily composed of Fe–O–Si. No significant difference in magnetic properties between the different Fe@SiO_2_ NPs samples was observed. (iii) After air exposure, whatever the thickness of the silica shell, the magnetization as well as the Fe(0) content of iron cores have greatly decreased, proving that the silica shell is not adapted to protect iron cores from their oxidation under air. (iv) After water exposure, the silica shell is drastically modified. Iron II or III species were produced due to the probable diffusion of water and then migrated into silica to form segregated paramagnetic species (in identical proportion regardless of the silica shell thickness). However, the comparative study between sample 2 and sample 4 shows that a greater thickness of shell allows limiting more effectively the fall of the magnetization of the iron cores during water exposure.

#### Heating power

**Fig. 8:**
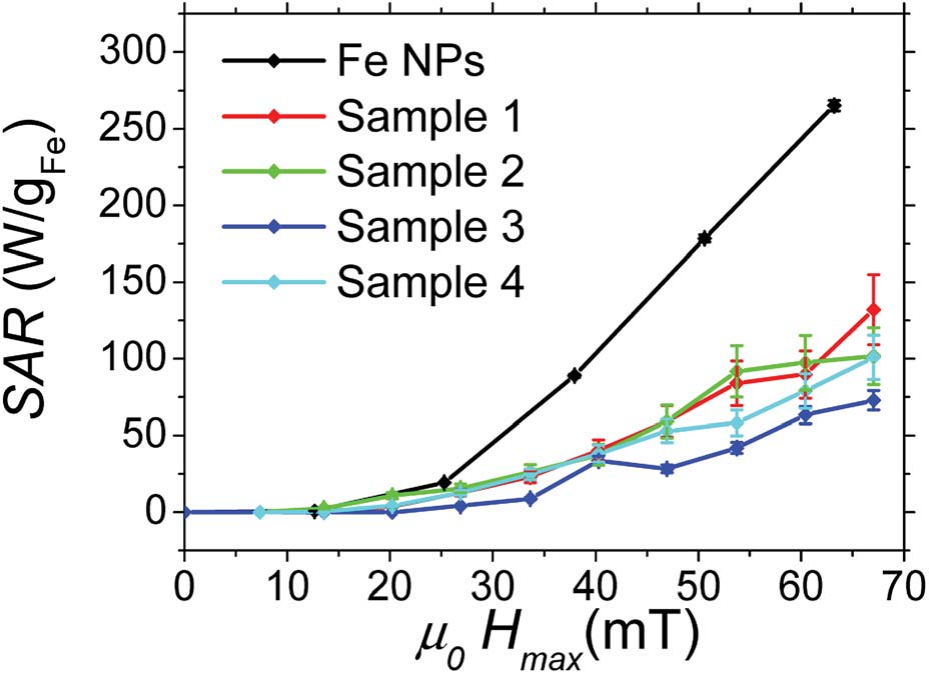
SAR values for each sample obtained from calorimetric measurements under AMF at 93 kHz and an amplitude range from 0 to 70 mT.

**Fig. 9:**
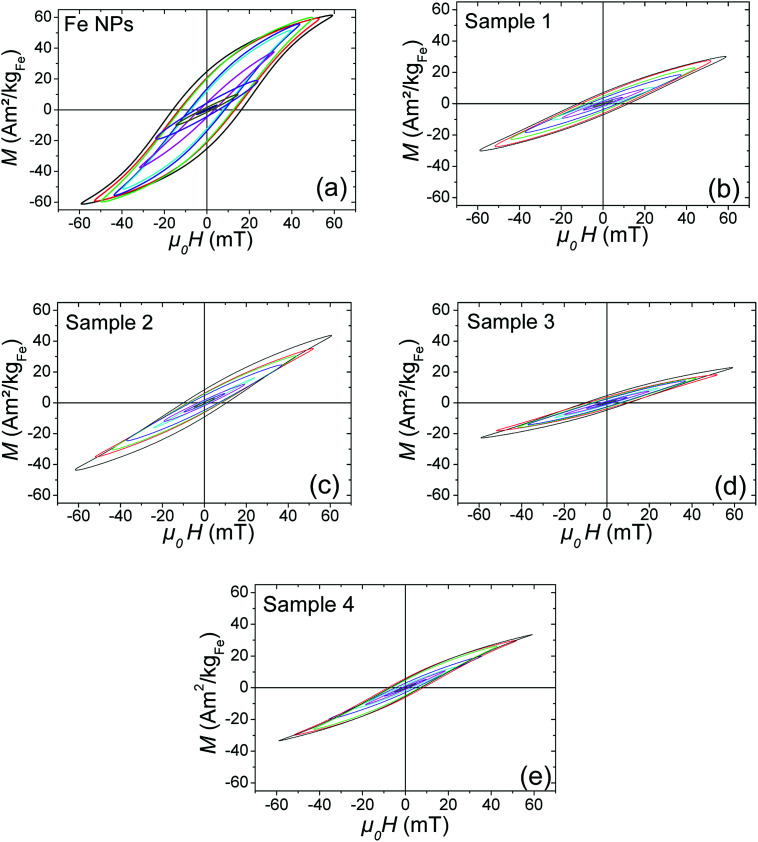
Magnetic hysteresis loops measured at different magnetic field amplitude: (a) Fe NPs, (b) sample 1, (c) sample 2, (d) sample 3, and (e) sample 4.

To quantify the heating power (SAR) of each sample (containing 10 to 30 mg of NPs in 0.5 mL of mesitylene), a calorimetric measurement was carried out (see Magnetic hyperthermia in Experimental part). Moreover, high-frequency hysteresis loops for each sample were measured to characterize their magnetic properties during AMF application. The hysteresis loops were calibrated using the results of SAR calorimetric measurements (as detailed in Experimental part and ESI†). These two types of measurement were performed on samples 1, 2, 3, 4 and Fe NPs. The corresponding results are reported in Fig. 8 and 9, and in ESI†-Fig. 4.

Since SAR amplitude depends strongly on the amplitude of the applied magnetic field and frequency, a convenient way to compare the heating properties of MNPs measured under various experimental conditions is to calculate their intrinsic loss parameter (ILP), defined by the equation 
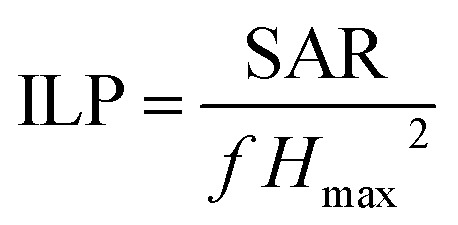
 with *H*_max_ the AMF maximum value (in A m^−1^), *f*, the AMF frequency (in Hz) and SAR the specific absorption rate (in W kg^−1^).^29^ The ILP is commonly expressed in nHm^2^ kg^−1^ (see Table 6). The evolution of ILP as a function of the magnetic field is shown in ESI†-Fig. 5. For the three Fe@SiO_2_ samples, ILP is rather independent of magnetic field amplitude so its average value is provided in Table 6. For the Fe NP samples, it increases significantly with the magnetic field amplitude, so the ILP range is not displayed in Table 6, but its evolution in function of magnetic field is provided in ESI†-Fig. 5. The origin of this phenomenon is discussed at the end of this section.

**Table 6:** ILP values for each coating samples. Uncertainly values were calculated with confidence interval of 99%. Evolution of ILP as a function of magnetic field amplitude of each sample is available in ESI

	ILP (nHm^2^ kg_Fe_^−1^)
Sample 1	0.40 (5)
Sample 2	0.43 (12)
Sample 3	0.24 (5)
Sample 4	0.31 (6)

As shown in Fig. 8, 9 and Table 6, samples 1, 2 and 4 present similar specific loss values, larger than the ones of sample 3. In this case, the iron content of the hybrid NPs is higher than in samples 1, 2, 4, and can influence the amplitude of magnetic interactions. SAR values are indeed very sensitive to the latter.^30^

The modest ILP values (in view of the results from the literature ref. 31) of the Fe@SiO_2_ NPs could be explained by the diameters of the cores. Using iron NPs with diameter in the range 16–20 nm, which displays much larger SAR values than the present one, could permit to increase these ILP values.^7^ However, the strong magnetization of such large MNPs tends to force their agglomeration in solution, impeding the coating process.

Finally, the Fe sample presents, at low magnetic field, heating and magnetic properties almost similar to the Fe@SiO_2_ samples. However, when the magnetic field is above 25 mT, Fe sample heating power becomes much larger than the other samples. Hysteresis loops in Fig. 9 show that this increase is associated with both a tilt of the hysteresis loops and an increase in remanence. Moreover, the minor loops at low magnetic field are not included any more in the loop at larger magnetic field, evidencing a change of regime. These features have been measured several times and are reproducible. They can be interpreted as a signature of the mobility of the free Fe NPs in solution during AMF application: above 25 mT, they self-organize in chains and/or have their anisotropy axis oriented along the magnetic field, which increases the ferrofluid susceptibility and remanence. Since ILP can be linked to magnetic susceptibility of ferrofluid (as detailed in ESI†), the increase of this last parameter as a function of magnetic field amplitude due to magnetic interaction could explain the ILP dependence on magnetic field amplitude for Fe NPs sample. The influence of the formation of chains of MNPs on heating properties has been reported by many groups.^8,32–34^ It is however the first time to our knowledge, that the signature of this formation (*i.e.* the increase of the ferrofluid susceptibility and remanence), is observed clearly with high-frequency hysteresis loop measurements. Moreover, in our case, a change of regime is observed, the formation of chains occurring only at large magnetic fields. Since the heating properties of Fe@SiO_2_ NPs and Fe NPs at low magnetic field are similar, the weaker heating power of the Fe@SiO_2_ NPs at large AMF compared to Fe NPs must not be interpreted as the signature of degraded properties due to the coating. The higher heating power of the Fe NPs is the consequence of their self-organization whereas the iron cores are immobilized inside the silica for Fe@SiO_2_ NPs during the magnetic field application. As example, the influence of NPs immobilization on their SAR decrease had been described for iron oxide NPs embedded in agarose or polyvinyl alcohol.^35^ In our case, silica can be seen as a more drastic freezing matrix, precluding any shift of the shelled iron NPs.

## Conclusions

This work provides a complete study of Fe@SiO_2_ composites by coupling XPS and magnetic measurements to describe their evolution under different atmospheres (argon, air, water) and to evaluate their hyperthermia properties. An original protocol has been developed in non alcoholic medium to coat iron NPs by silica, which remarkably allows the preservation of the magnetization of the iron cores once coated. If the silica layer formation induces a detectable increase of the ratio Fe(ii/iii)/Fe(0) at the interface with silica, it is limited to few atomic layers and is not large enough to have significant consequences on the magnetic properties. The study of the Fe@SiO_2_ exposed to air or to water has shown that magnetization could be interestingly preserved in water thanks to a thick silica layer but not in air, whatever the silica thickness. The water exposure leads to the formation of isolated paramagnetic species, which could be the result of the diffusion of water through the silica shell to form Fe(ii) or Fe(iii) species migrating then into the silica. Over the time, in water, the silica shell is progressively degraded leading to the total dissolution of the Fe@SiO_2_ composites after several months. Interestingly, by comparing hyperthermia measurements between Fe NPs and Fe@SiO_2_, we have been able to evidence the self-organization of the free Fe NPs, when a large amplitude magnetic field is applied. High-frequency hysteresis loop measurements allowed us to observe for the first time the signature of the formation of Fe NPs chains, evidenced by the increase of the ferrofluid susceptibility and remanence. This induces thus an increase of heating power, this phenomenon being precluded when the MNPs are immobilized.

## Conflicts of interest

There are no conflicts to declare.

## Supplementary Material

RA-008-C8RA06075D-s001

## References

[cit1] Meffre A., Mehdaoui B., Connord V., Carrey J., Fazzini P. F., Lachaize S., Respaud M., Chaudret B. (2015). Nano Lett..

[cit2] Kefeni K. K., Msagati T. A. M., Mamba B. B. (2017). J. Mater. Sci. Eng. B.

[cit3] Colombo M., Carregal-Romero S., Casula M. F., Gutiérrez L., Morales M. P., Böhm I. B., Heverhagen J. T., Prosperi D., Parak W. J. (2012). Chem. Soc. Rev..

[cit4] Lim E.-K., Kim T., Paik S., Haam S., Huh Y.-M., Lee K. (2015). Chem. Rev..

[cit5] Lacroix L.-M., Delpech F., Nayral C., Lachaize S., Chaudret B. (2013). Interface Focus.

[cit6] Rollet A.-L., Neveu S., Porion P., Dupuis V., Cherrak N., Levitz P. (2016). Phys. Chem. Chem. Phys..

[cit7] Mehdaoui B., Meffre A., Carrey J., Lachaize S., Lacroix L.-M., Gougeon M., Chaudret B., Respaud M. (2011). Adv. Funct. Mater..

[cit8] Mehdaoui B., Tan R. P., Meffre A., Carrey J., Lachaize S., Chaudret B., Respaud M. (2013). Phys. Rev. B.

[cit9] Bohara R. A., Thorat N. D., Pawar S. H. (2016). RSC Adv..

[cit10] Piñeiro Y., Vargas Z., Rivas J., López-Quintela M. A. (2015). Eur. J. Inorg. Chem..

[cit11] Tao C., Zhu Y. (2014). Dalton Trans..

[cit12] Tian Z., Yu X., Ruan Z., Zhu M., Zhu Y., Hanagata N. (2018). Microporous Mesoporous Mater..

[cit13] Yao X., Niu X., Ma K., Huang P., Grothe J., Kaskel S., Zhu Y. (2017). Small.

[cit14] Yu X., Zhu Y. (2016). Sci. Technol. Adv. Mater..

[cit15] Zhu Y., Tao C. (2015). RSC Adv..

[cit16] Carrey J., Medhaoui B., Respaud M. (2011). J. Appl. Phys..

[cit17] El-Hawi N., Nayral C., Delpech F., Coppel Y., Cornejo A., Castel A., Chaudret B. (2009). Langmuir.

[cit18] Sonmez M., Georgescu M., Alexandrescu L., Gurau D., Ficai A., Ficai D., Andronescu E. (2015). Curr. Pharm. Des..

[cit19] Lee H., Ryu J., Kim M., Im S. S., Kim I. S., Sohn D. (2016). Radiat. Phys. Chem..

[cit20] Vivero-Escoto J. L., Huxford-Phillips R. C., Lin W. (2012). Chem. Soc. Rev..

[cit21] Narita A., Nakab K., Chujo Y. (2009). Colloids Surf. A.

[cit22] Vogt C., Toprak M. S., Muhammed M., Laurent S., Bridot J.-L., Müller R. N. (2010). J. Nanopart. Res..

[cit23] Shirley D. A. (1972). Phys. Rev. B.

[cit24] Biesinger M. C., Payne B. P., Grosvenor A. P., Lau L. W. M., Gerson A. R., St R., Smart C. (2011). Appl. Surf. Sci..

[cit25] Scofield J. H. (1976). J. Electron Spectrosc. Relat. Phenom..

[cit26] Connord V., Mehdaoui B., Tan R. P., Carrey J., Respaud M. (2014). Rev. Sci. Instrum..

[cit27] Wilson D., Langell M. A. (2014). Appl. Surf. Sci..

[cit28] Okada K., Kameshima Y., Yasumori A. (1998). J. Am. Ceram. Soc..

[cit29] Wildeboer R. R., Southern P., Pankhurs Q. A. (2014). J. Phys. D: Appl. Phys..

[cit30] Tan R. P., Carrey J., Respaud M. (2014). Phys. Rev. B.

[cit31] Ludwig R., Stapf M., Dutz S., Muller R., Teichgraber U., Hilger I. (2014). Nanoscale Res. Lett..

[cit32] Mehdaoui B., Meffre A., Lacroix L.-M., Carrey J., Lachaize S., Goujeon M., Respaud M., Chaudret B. (2010). J. Magn. Magn. Mater..

[cit33] Saville S. L., Qi B., Baker J., Stone R., Camley R. E., Livesey K. L., Ye L., Crawford T. M., Mefford O. T. (2014). J. Colloid Interface Sci..

[cit34] Klokkenburg M., Erńe B. H., Meeldijk J. D., Wiedenmann A., Petukhov A. V., Dullens R. P. A., Philipse A. P. (2006). Phys. Rev. Lett..

[cit35] Ludwig R., Stapf M., Dutz S., Müller R., Teichgräber U., Hilger I. (2014). Nanoscale Res. Lett..

