# Supplementary material for: Silica coated iron nanoparticles: synthesis, interface control, magnetic and hyperthermia properties
Source: RSC Adv. 2018 Sep 17;8(56):32146–56. doi: 10.1039/c8ra06075d (PMC9085846; doi:10.1039/c8ra06075d)
Supplement: RA-008-C8RA06075D-s001 [file RA-008-C8RA06075D-s001.pdf]

Electronic Supplementary Material (ESI) for RSC Advances.
This journal is © The Royal Society of Chemistry 2018

Electronic Supplementary Information.

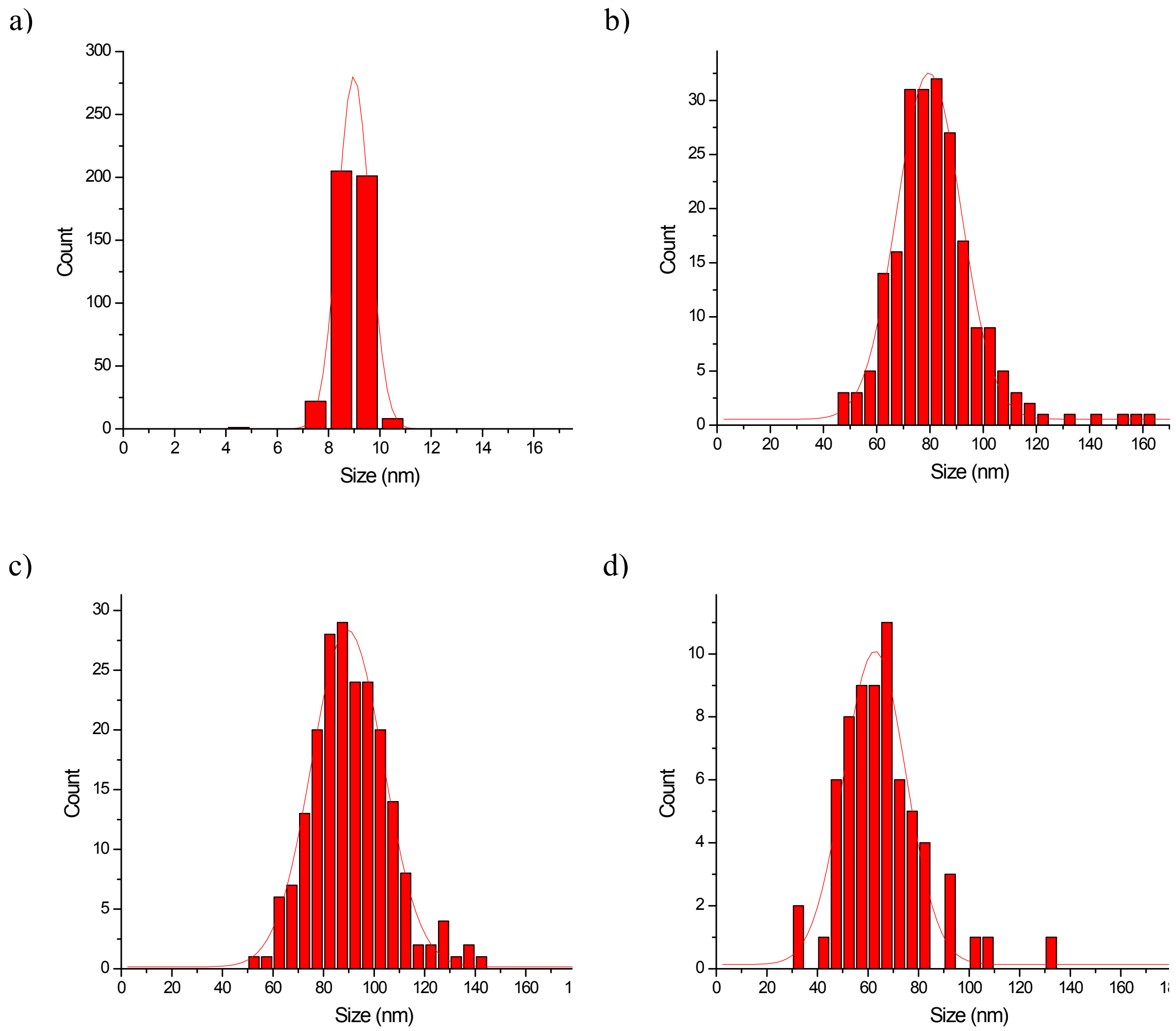

ESI-Fig. 1: Size histograms of the (a) the pristine Fe NPs, (b) sample 1, (c) sample 2 and (d) sample 3

ESI-Table 1: Relative proportions of Fe(0) and Fe(II/III), Fe(II/III)/Fe(0) ratio calculated from XPS analyses for Fe nanoparticles and the same material exposed to air and water

	**Fe(0) (rel. %)**	**Fe(II/III) (rel. %)**	**Fe(II/III)/Fe(0)**
Fe NPs	40.8	59.2	1.5
Fe NPs-air	9.2	90.8	9.9
Fe NPs-H_2O	21.5	78.5	3.7

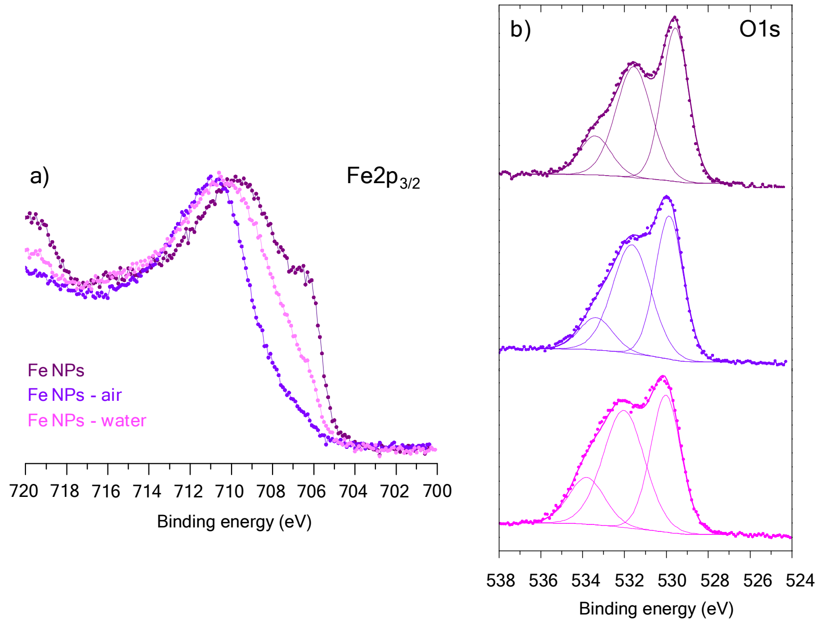

ESI-Fig. 2: a) $Fe2p_{3/2}$, b) O1s XPS core peaks of Fe nanoparticles before and after air and water exposure

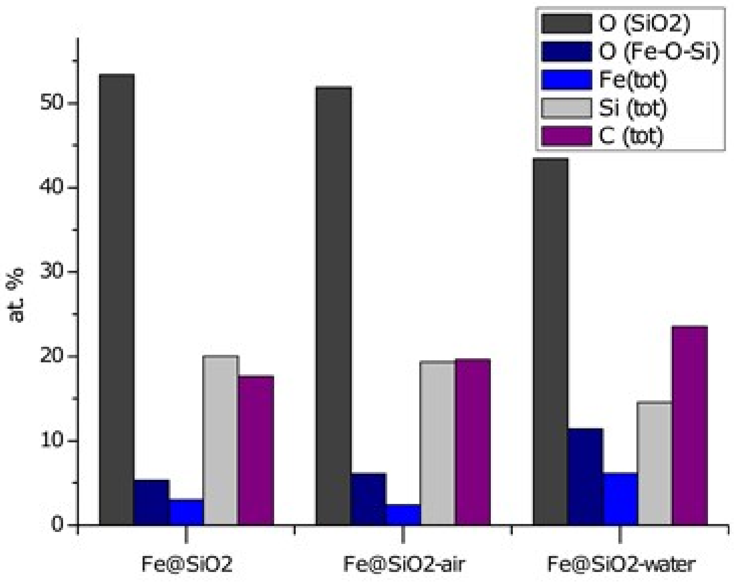

ESI-Fig. 3: Atomic percentages of $O(SiO_2)$, O(Fe-O-Si), Fe(tot), Si(tot) and C(tot) of $Fe@SiO_2$ and the same materials exposed to air and water.

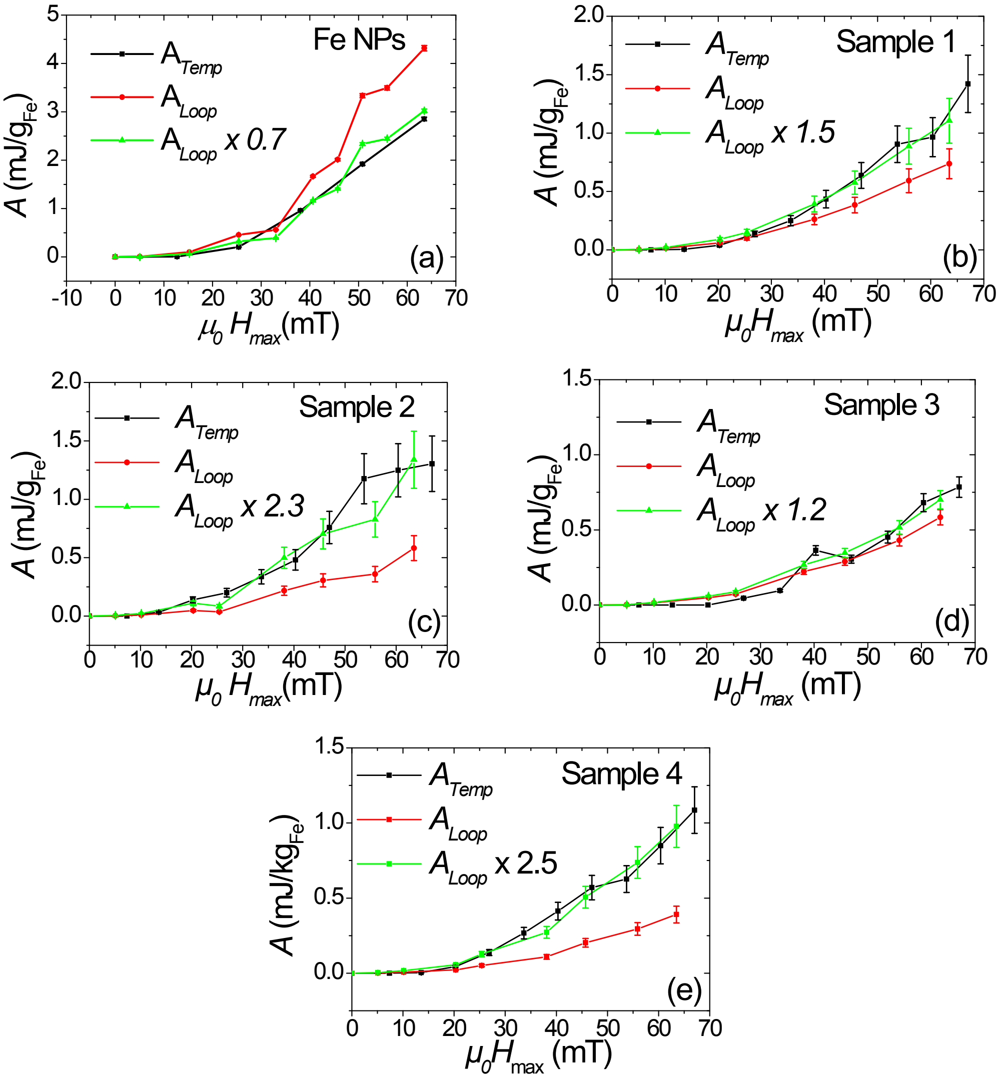

ESI-Fig. 4: Comparison of A_{Temp} (black squares) and A_{loop} (red diamonds) to determine x_{corr} factor (green triangles represent $A_{loop}\,x_{corr}$ curve) for a) Fe NPs (x_{corr}=0.7), b) sample 1 (x_{corr}=1.5), c) sample 2 (x_{corr}=2.3), d) sample 3 (x_{corr} =1.2), and e) sample 4 (x_{corr}=2.5). Error bars are measurement uncertainty propagation of the weight content of iron in each sample.

Calibration of high-frequency hysteresis loop measurement:

From calorimetric measurements, the SAR values of the NPs are obtained for one frequency of AMF (93 kHz in the present case).Dividing the specific absorption rate (SAR*)* values by the magnetic field frequency gives the specific losses A, noted as A_{Temp} for this method (in mJ per gram of pure iron).

With the high frequency hysteresis loop measurement, specific losses A_{Loop} of each sample are determined by calculating the hysteresis loop area. Such a method is quantitative only if the samples are a perfectly homogeneous colloidal solution, which is not the case here,

since the NPs have a tendency to precipitate (within 0.5 ml of mesitylene), in particular the Fe@SiO_2 samples.

To obtain the hysteresis loops with the correct magnetization scale, the series of measurements for a given sample were multiplied by a correcting factor to be in adequation with the calorific method (see ESI-Fig.4). Both methods are complementary: the first one allows quantification of specific losses of each sample and the second method gives more information about magnetic properties thanks to the complete hysteresis loop.

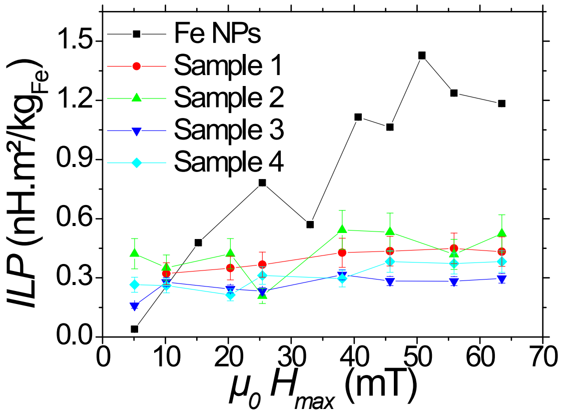

ESI-Fig. 5: *ILP* calculation as a function of magnetic field amplitude. Error bars are propagation of measurement uncertainty on the weight content of iron in each sample.

Discussion on the domain of validity of *ILP*:

As noted by Wildeboer et al.,[1] heat power (*SAR*) produced by magnetic NPs depends of intrinsic and extrinsic factors. Intrinsic factors are linked to physical and magnetic properties of magnetic NPs. Extrinsic factors are frequency and amplitude of AMF applied and could vary from one experiment to another. Therefore to compare heat efficiency of several magnetic NPs, under magnetic hyperthermia conditions, it is important to only consider intrinsic factors. For this reason, *ILP* seems to be a suitable candidate but its intrinsic character is only true within a restricted domain of validity.

At low value of magnetic field amplitude, this parameter is constant and directly proportional to (œá") the imaginary component of the complex susceptibility.[2] Indeed, in this regime where the linear response theory (LRT) is valid, hysteresis loops of nanoparticles are ellipses whose area (*A*), and so specific losses, can be calculated by this expression:

$$A = \text{œÄ}\, H_{max}^{2}\, \text{œá"}$$

Let us remember that:

$$SAR = A/f \text{ and } ILP = {}^{SAR}/fH_{max}^{2}$$

Thus :

$$ILP = \frac{A}{H_{max}^{2}} = \text{œÄ œá''}$$

However, the last expression is only true with low field strength. Beyond a certain value of H_{max}, when $\text{¬µ}_0 H_{max} M_s V/k_B T\text{,â•}1$, with M_s the saturation magnetization and V the NPs volume,[3] hysteresis loops do not keep their ellipse-shape. *ILP* varies when H_{max} increases and is no more linked to magnetic susceptibility of the nanomaterial. Therefore a proper *ILP* calculation is only achieved within the LRT range validity. Moreover, it is important to note *ILP* can also depend on magnetic field amplitude for some cases of interacting NPs assembly, as observed in ESI Fig.5, where magnetic susceptibility increases with magnetic field (see Figure 9-a).

Determination of M_{ferro} and M_{para} contributions:

The non-saturation of the magnetic hysteresis loops at high field, is consistent with the formation of paramagnetic species, such as electrically insulated Fe(II) and/or Fe(III). To determine their proportion ($\%_{para}$), and their contribution (M_{para}) on the magnetic signal measured by VSM (M_{VSM}) at 4 K, the latter can be expressed as follows:

$$M_{VSM} = \%_{para}M_{para} + \%_{ferro}M_{ferro} \text{ with } \%_{para} + \%_{ferro} = 1$$

M_{ferro} is the ferromagnetic contribution to the magnetic signal. It can thus be deduced that

$$M_{ferro} = \frac{M_{VSM} - \%_{para}M_{para}}{1 - \%_{para}}$$

We assumed that the paramagnetic magnetization M_{para} comes from magnetically independent Fe(II) or Fe(III) species so that:

$$M_{para} = \frac{N_A}{M_{Fe}} g\text{¬µ}_B J_T B_{J_T}\left(\frac{g\text{¬µ}_B J_T \text{¬µ}_0 H}{k_B T}\right)$$

where N_A and M_{Fe} are, respectively, Avogadro number and atomic molar mass of iron (around 55.8 g/mol). μ_B is the Bohr magneton, k_B the Boltzmann constant, T the temperature, $\mu_0 H$ the applied magnetic field, $B_{J_T}(x)$ the Brillouin function, J_T the atomic kinetic moment and g the Landé factor. For Fe(II), $g = 1.5$ and $J_T = 4$; for Fe(III), $g = 2$ and $J_T = 2.5$.

Determination of $\%_{para}$ was carried out by separating the magnetic signal into one contribution which saturates at large magnetic field and a second non-saturating one. The former was attributed to M_{ferro} and the latter to M_{para}. It was thus assumed that the diamagnetic contribution on M_{VSM} is negligible. The saturation value of the M_{ferro} signal is called $M_{\mathrm{s,ferro}}$.

1 R R Wildeboer, P Southern and Q A Pankhurst. *J. Phys. D: Appl. Phys.,* 2014, **47**, 495003.

2 V. Connord. B. Mehdaoui. R. P. Tan. J. Carrey. and M. Respaud. *AIP Rev. Sci. Instrum.,* 2014, **85**, 093904.

3 J. Carrey. B. Mehdaoui. and M. Respaud. *J. App. Phys.,* 2011, **109**, 083921.
